# Assessing User Engagement of an mHealth Intervention: Development and Implementation of the Growing Healthy App Engagement Index

**DOI:** 10.2196/mhealth.7236

**Published:** 2017-06-29

**Authors:** Sarah Taki, Sharyn Lymer, Catherine Georgina Russell, Karen Campbell, Rachel Laws, Kok-Leong Ong, Rosalind Elliott, Elizabeth Denney-Wilson

**Affiliations:** ^1^ University of Technology Sydney Sydney Australia; ^2^ Centre for Obesity Management and Prevention Research Excellence in Primary Health Care Sydney Australia; ^3^ University of Sydney Sydney Australia; ^4^ Deakin University Victoria Australia; ^5^ Latrobe University Melbourne Australia

**Keywords:** mHealth, social medium, infant obesity, infant development, children, infants, practitioners, primary healthcare

## Abstract

**Background:**

Childhood obesity is an ongoing problem in developed countries that needs targeted prevention in the youngest age groups. Children in socioeconomically disadvantaged families are most at risk. Mobile health (mHealth) interventions offer a potential route to target these families because of its relatively low cost and high reach. The Growing healthy program was developed to provide evidence-based information on infant feeding from birth to 9 months via app or website. Understanding user engagement with these media is vital to developing successful interventions. Engagement is a complex, multifactorial concept that needs to move beyond simple metrics.

**Objective:**

The aim of our study was to describe the development of an engagement index (EI) to monitor participant interaction with the Growing healthy app. The index included a number of subindices and cut-points to categorize engagement.

**Methods:**

The Growing program was a feasibility study in which 300 mother-infant dyads were provided with an app which included 3 push notifications that was sent each week. Growing healthy participants completed surveys at 3 time points: baseline (T1) (infant age ≤3 months), infant aged 6 months (T2), and infant aged 9 months (T3). In addition, app usage data were captured from the app. The EI was adapted from the Web Analytics Demystified visitor EI. Our EI included 5 subindices: (1) click depth, (2) loyalty, (3) interaction, (4) recency, and (5) feedback. The overall EI summarized the subindices from date of registration through to 39 weeks (9 months) from the infant’s date of birth.

Basic descriptive data analysis was performed on the metrics and components of the EI as well as the final EI score. Group comparisons used *t* tests, analysis of variance (ANOVA), Mann-Whitney, Kruskal-Wallis, and Spearman correlation tests as appropriate. Consideration of independent variables associated with the EI score were modeled using linear regression models.

**Results:**

The overall EI mean score was 30.0% (SD 11.5%) with a range of 1.8% - 57.6%. The cut-points used for high engagement were scores greater than 37.1% and for poor engagement were scores less than 21.1%. Significant explanatory variables of the EI score included: parity (*P*=.005), system type including “app only” users or “both” app and email users (*P*<.001), recruitment method (*P*=.02), and baby age at recruitment (*P*=.005).

**Conclusions:**

The EI provided a comprehensive understanding of participant behavior with the app over the 9-month period of the Growing healthy program. The use of the EI in this study demonstrates that rich and useful data can be collected and used to inform assessments of the strengths and weaknesses of the app and in turn inform future interventions.

## Introduction

Mobile phone ownership is widespread in Australia and internationally [1,2] and many people use their phone to gain information, browse websites, and use apps [1,2]. Ownership of mobile phones is high across all socioeconomic groups and the mobile phone is a promising tool for delivery of behavior change interventions [3,4]. A mobile phone app was used to provide information and support to parents regarding infant feeding for the Growing healthy program in Australia [5].

Capturing the attention of an app user is clearly paramount to the app’s potential effectiveness for behavior change. To be successful, apps must continuously and actively engage the user. User engagement refers to the quality of the user experience, the positive aspects of their interaction, and their desire to use the app over longer periods of time or repeatedly [6]. A recently published review on digital behavior change interventions identified that content and delivery, the setting in which the intervention is used, the demographic, and the targeted behavior influences engagement [7]. Furthermore, the Medical and Research Council (MRC) framework emphasized the importance to utilize theoretical models for the development of effective interventions [8]. This was further supported by the findings in a review which explored the effectiveness of mobile phone apps targeting health behaviors [9]. They identified that interventions which utilized health behavior models were more likely to have an impact.

Engagement with technology is inherently complex and multifaceted in its nature and it may be mediated by factors such as family, community, culture, and context [10]. O’Brien and Toms [11] posit that engagement is not static, but a process with four distinct stages: (1) point of engagement, (2) period of engagement, (3) disengagement, and (4) reengagement. Thus, a user’s engagement is considered to be operating over a continuum and this may vary within a session and over long time periods [11]. User engagement and its measurement can be either short or long term, with long term engagement reflecting the degree of involvement a user has with the system (eg, an app) over time [12]. There have been multiple approaches to the measurement of user engagement, reflecting the many elements considered to comprise engagement. These include users’ physical participation in a specific target behavior and behavior in virtual spaces (eg, frequency of access), although it is the user’s psychological state and perceived experience that is most relevant to engagement [10].

Large scale quantitative measures of engagement rely on Web analytics which provide the opportunity to measure behavioral aspects of engagement. Some examples of data that can be collected, but is not exhaustive to, includes frequency of access to the app, page views, push notifications opened, and average time spent on a page [13]. These metrics unlike other engagement measures based on subjective questionnaires and psychological testing can be applied to the study population with no respondent burden. Web analytics provide insight via these proxy measures about the dynamics of participant engagement and its relationship with app effectiveness. They also provide insights regarding areas for app improvements, lower participant attrition, and in turn increased intervention exposure [14,15].

It has been suggested that Web analytics measures can be classed into three main dimensions of engagement: popularity, activity, and loyalty [6]. To achieve a more in-depth understanding of consumer behaviors and their influencers, “engagement indices,” accounting for these three dimensions of engagement, have been used to calculate the users’ overall interaction with Web-based technologies [16,17]. Engagement indices provide quantitative evidence regarding the strengths and weaknesses of website and app features to optimize participant engagement and sustainable long-term use of the app.

Little work has been done in the mHealth arena with respect to the conceptualization and measurement of user engagement [7,18,19]. The work done has been mainly around apps for patient engagement of those with chronic disease or around public health and behavior change such as increased physical activity and weight loss [20-22]. Few mHealth programs comprehensively use the available data to analyze participant engagement or to consider its associations with primary outcomes [18,20,21,23]. This paper describes the development of a fit-for-purpose engagement index (EI) based on Web metrics that allows large scale implementation. The EI reported in this paper was developed for the Growing healthy program which used a mobile phone app [5] to provide information and support to parents regarding infant feeding. We provide a rationale and description of the development of an EI to measure participants’ behavior utilizing the Growing healthy app; describe the assignment of cut-points for poorly, moderately, or highly engaged users; and investigate determinants effecting participants’ engagement with the app.

## Methods

### Growing Healthy Feasibility Study

The Growing healthy program utilized a quasi-experimental design aimed to support parents of young infants with healthy infant feeding behaviors. To enhance intervention effectiveness, the behavior change wheel model [24], as well as the mode of delivery, content, and quality of the program were considered during the development phase.

Eligible participants were offered to use the Growing healthy app and could choose to receive 3 tailored push notifications through the app each week of the intervention (9 months of the baby’s age). Although, midway through the intervention implementation period, participants were also sent a weekly email due to identifying technological issues with receiving and opening push notifications. The weekly emails included the same messages as the push notifications sent each week. Participants who did not own a phone that was compatible with the app were offered access to the Growing healthy website and were sent 3 text messages. Details of the study has been published previously [5]. The focus of recruitment was parents from socioeconomically disadvantaged regions and resulted in 300 participants. Recruitment was conducted via health practitioners, face to face, or Web-based methods. Eligibility criteria included: (1) expectant parents (30+ weeks gestation) or parents with an infant less than 3 months of age, (2) literate in English, (3) living in Australia, (4) 18 years or older, and (5) ownership of any type of mobile phone or Internet access. Further details of the recruitment process and outcomes have been published elsewhere [25]. As Growing healthy was a feasibility study, the sample size was tailored to logistical limitations of the time and funds available to support recruitment. The EI scoring was only performed for participants’ data when the participant registered for the Growing healthy app, activated and accessed the app at least once, and used the app and opened push notifications or weekly emails of the Growing healthy program. The focus of this paper was to report the EI for the intervention group.

Study participants completed 3 quantitative surveys: (1) baseline (T1) (infant age ≤3 months), (2) infant aged 6 months (T2), and (3) infant aged 9 months (T3). The surveys included demographic and infant feeding behavior questions. Participants’ use of the app was captured and the data was used to develop the EI and evaluate the Growing healthy program.

### Engagement Index

**Figure 1:**
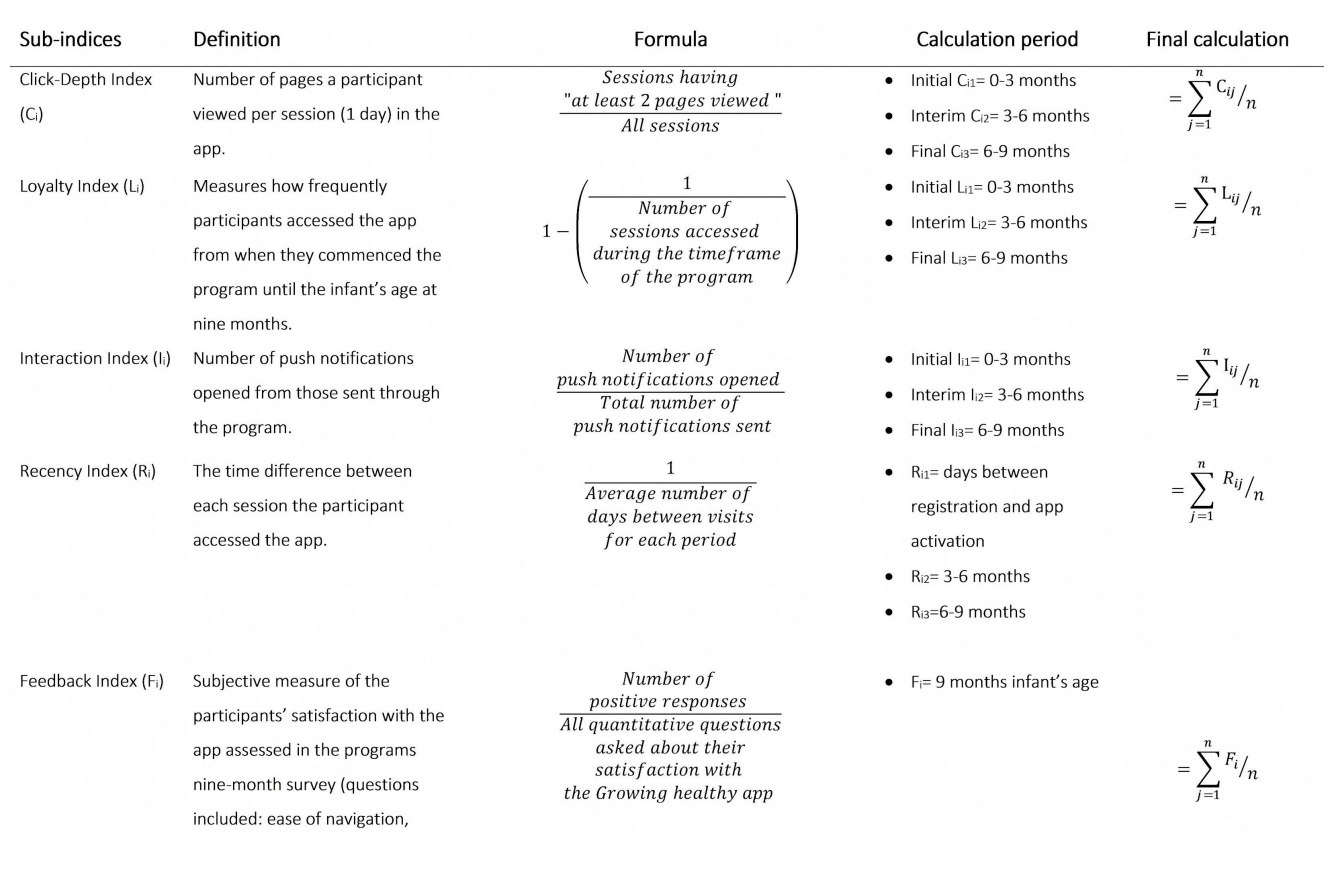
The definitions of the subindices for the engagement index designed for the Growing healthy program where i=ith person and j=jth time period and n=3 for Ci, Li, Ii, and Ri (sum of calculation period) and n=37 for Fi.

The Web Analytics Demystified visitor EI [16] was adapted to develop a composite measure of engagement for Growing healthy app users. This index was chosen because a detailed description on how to develop and apply it was available. The original index comprised 7 subindices which measured: (1) click depth, (2) loyalty, (3) recency, (4) interaction, (5) feedback, (6) brand, and (7) duration index. Figure 1 presents the adapted term-definitions of subindices included in this study (see Multimedia Appendix 1 for questions). All but 2 subindices (brand index and duration index) from the Web Analytics Demystified visitor EI were available from the app database collected in this study. Although measuring all indices is ideal, the Web Analytics Demystified visitor EI protocol emphasized that the calculation can be adapted to suit the program based on data collected [16]. The developed EI provided a score for each participant that measured their overall engagement with the app against a predetermined criteria. The time frame under consideration was from date of registration to 39 weeks (9 months) from the participants’ infant’s date of birth.

Metrics needed to calculate the subindices (outlined in Figure 1) were identified and extracted from the Growing healthy app database. The key metrics collected included “session duration,” “page views per session,” and “number of push notifications opened.” Furthermore, subjective markers such as feedback and satisfaction captured at the T3 survey (9 month of the baby’s age) was also used to calculate the EI score.

Equal weight for each of the subindices was assigned to the overall EI score so that each element was equally important in contributing to the measurement of engagement. Four of the subindices were calculated using app data. The feedback index was informed using responses to the 9-month survey (T3) feedback questions. The final formula used to calculate the EI incorporated click depth, loyalty, recency, interaction, and feedback subindices (see equation 1). The EI was then converted to a value between 0 and 100.

Equation 1: Engagement index formula

EI=∑(C
_i_+L
_i_+I
_i_+R
_i_+F
_i_)×100

where EI is engagement index, C_i_ is click depth index, L_i_ is loyalty index, I_i_ is interaction index, R_i_ is recency index, and F_i_ is feedback index.

The calculation for each subindex except the feedback index (data were only collected at the end of the program) was done for three time periods, including initial (0-3 months), interim (3-6 months), and final (6-9 months) and were then averaged. This grouping of time periods was chosen because there was an initial intense use of the app followed by infrequent participant use toward the end of the 9-month program. A detailed explanation for the calculation of each subindex follows.

#### Click Depth Index (Ci)

The number of pages a participant viewed the app in each access session over the total number of sessions in each time period formed the basis of this subindex. Two metrics were used in the calculation of C_i_: the number of sessions in the time period and number of pages viewed per session. A threshold of the number of pages viewed per session was applied. There is no benchmark of an effective click depth, that is, “dose” of the interaction in the mHealth environ. Based on the data collected, the median value of 2 pages per session was used as the threshold. The overall score of C_i_ was the average of each time period calculation: C_i1_, C_i2_, and C_i3_.

#### Loyalty Index (Li)

This subindex was based on the frequency of app access throughout the 9-month program. L_i_ was the reciprocal of the number of sessions in each time period. The total score was dependent on when participants activated the app. The overall score of L_i_ was the average of each time period calculation: L_i1_, L_i2_, and L_i3_.

#### Interaction Index (Ii)

The number of push notifications opened versus total sent throughout the 9-month program formed the basis of this subindex. Interaction Index was the total number of push notifications opened divided by the number sent in the time period. This was calculated for 3 month time intervals of the infant’s age according to when the participant activated the app until the infant reached 9 months of age. The overall score of I_i_ is the average of each time period calculation: I_i1_, I_i2_, and I_i3_.

#### Recency Index (Ri)

The number of days between each session was the basis of the recency index. The R_i_ was calculated for three different time points: (1) the number of days elapsed from registration to when the participant first accessed the app (R_i1_), (2) the average number of days between sessions when the participant accessed the app between 3 to 6 months (R_i2_), and (3) 6 and 9 months (R_i3_). The data were transformed by taking the reciprocal of each R_i1_ to R_i3_. The overall score of R_i_ was the average of each time period calculation: R_i1_, R_i2_, and R_i3_.

#### Feedback Index (Fi)

This subindex was a self-reported measure of participant satisfaction with the app, which was captured in the 9-month survey (T3). Constructive feedback was scored positively as 1 and negatively as 0. The 9-month survey included 37 questions which formed the basis of F_i_. Each question (Multimedia Appendix 1) used a 5-point Likert scale response ( “strongly agree” to “strongly disagree” and “didn’t use”). The responses were dichotomized as either 1 or 0 according to whether they answered an extreme positive response or not; for example: strongly agree=1, agree=0, neither here nor there=0, disagree=0, strongly disagree=0, and didn’t use=0. Extreme positive scoring was reversed on the Likert scale for questions worded negatively. Although only app users were eligible for this study, some app users reported using the website rather than the app in the T3 survey (n=15) and thus were not asked the feedback questions. The EI total score for these participants were averaged across the 4 subindices that data were available. In addition, a number of participants (n=102) did not complete the T3 survey. For these participants F_i_ was zero and the EI was averaged across the 5 subindices.

### Statistical Analysis

Basic descriptive data analysis was performed on the metrics and components of the EI as well as the final EI score. To analyze the EI scores, cut-off points were developed based on the distribution of the total samples’ EI scores using quartiles. Participants were then categorized as either poorly, moderately, or highly engaged. This method was chosen as there were no existing mHealth interventions that utilized an EI and categorized participants’ engagement based on app use.

Group comparisons between poorly, moderately, or highly engaged participants were then conducted using *t*-tests, analysis of variance (ANOVA), Mann-Whitney, Kruskall-Wallis, and Spearman correlation tests were used as appropriate. Consideration of independent variables associated with the EI score were modeled using linear regression models.

The following variables were dichotomized for analysis including:

- Education level: university degree (“degree” or “higher degree”) or no university (“high school education or less,” “trade certificate,” or “diploma”)
- Employment status: working or studying (“full or part-time,” “casual paid work,” and “full or part-time studying”) or not in labor force (“keeping house and/or raising children full-time” and “unemployed or laid off”)
- Gross household income: below average (“Aus $1-$119 per week,” “Aus $120-$299 per week,” “Aus $300-$599 per week,” “Aus $600-$799 per week,” “Aus $800-$999 per week”) average (“Aus $1000-$1499 per week”), above average (“Aus $1500-1999 per week”), or higher income (“Aus $2000 or more per week”)
- Marital status: relationship (“married,” “living in a defacto relationship”) or single (“separated,” “divorced,” “widowed,” “never married”)
- Recruitment method: practitioner, Web-based, or family or friends
- Device type: android or iOS
- System type: app only or both app and email

Other independent variables considered included mother’s age, country of birth, as well as infant’s age at the start of the program, their birth weight, and feeding status at baseline. All analyses were performed using used IBM SPSS Version 23.0.

## Results

Of the 300 Growing healthy participants who completed the baseline survey, 75.0% (225/300) met the inclusion criteria for this study. The average age of participants was 30 years, with 62.2% (186/300) being first time parents, 97.0 % (291/300) living with their partner, and 84.0% (252/300) being full-time carers of the infant. The infants’ were on average 6.9 weeks old when registration occurred and 56.4 %( 169/300) were breastfed.

**Figure 2:**
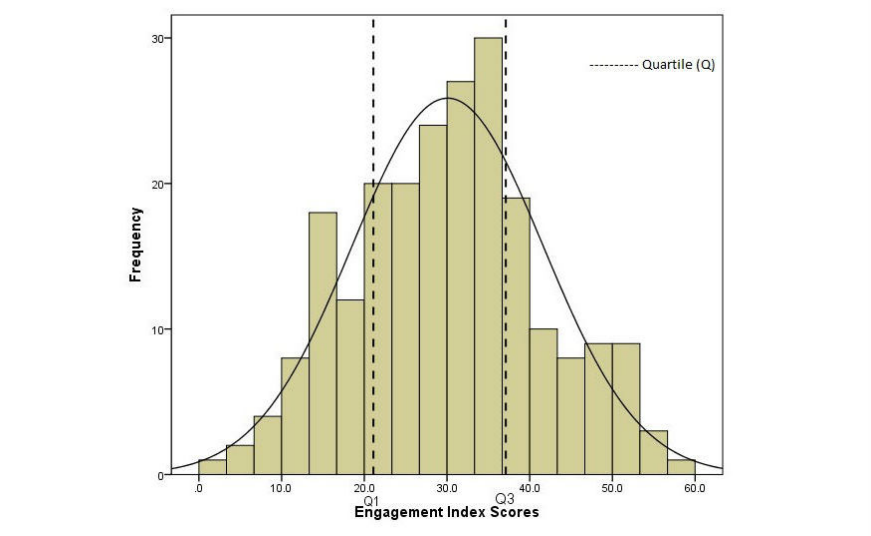
Overall engagement index scores distribution.

The EI score had a distribution that was not statistically significant as evidenced by nonsignificant Kolomogorov Smirnov (KS) test at *P* value of .05 and a standard error of skewness (SES) between >−1.96 and <1.96 (SES=0.58). The mean EI score was 30.0% (SD 11.5%) and ranged between 1.8% to 57.6% (see Figure 2). The interquartile ranges were used for categorization, where: (1) poor engagement for scores less than or equal to 21.1% (≤Q1), (2) moderate engagement if scores were between 21.1% and 37.1% (Q1-Q3), and (3) high engagement if score were greater than or equal to 37.1%(≥Q3).

Three variables were significantly associated with high engagement in univariate analysis (see Table 1 for details). Participants most likely to be classed as having high engagement were first time parents (primiparous), who used both the app and opened weekly emails, and had joined the program with a younger infant and were part of the program for longer. Approximately 64% of participants with higher level education (university degree) were classed as having high engagement compared with 55.3% of those with lower levels of education (no university). Additional demographic descriptors for the different engagement levels are shown in Table 1. Engagement index cut-points for scores: poor engagement ≤21.1%, moderate engagement=21.1%-37.1%, and high engagement ≥37.1%. Variables are based on data provided at baseline or T1 (age ≤3 months).

**Table 1:** Characteristics of growing healthy participants based on engagement index level (n=255).

Variables		Poor engagement (n=56) 15.1 (SD 4.6)	Moderate engagement (n=113) 30.0 (SD 4.3)	High engagement (n=56) 45.0 (SD 5.5)	*P* value
**Participant characteristics**					
	Age (years)^a^, mean (SD)	30.3 (4.4)	30.5 (4.4)	30.6 (4.5)	.61
	Education (no university)^b^, n (%)	31 (55)	61 (54)	20 (36)	.11
	Income (higher income)^c^, n (%)	14 (25)	36 (31.8)	13 (23.2)	.70
	Marital status (relationship)^b^, n (%)	54 (96)	110 (97.3)	53 (95)	.59
	Employment status (not in labor force)^b^, n (%)	51 (91)	97 (85.8)	45 (80)	.14
	Parity (Primiparous)^b^, n (%)	29 (52)	70 (61.9)	41 (73)	.004^d^
	Recruitment method (Practitioner)^c^, n (%)	24 (48)	52 (47.9)	30 (48)	.06
	Device type (iOS)^b^, n (%)	38 (68)	87 (72.5)	36 (64)	.73
	System type (both app & email users)^b^, n (%)	26 (46)	82 (72.5)	52 (93)	<.001^d^
**Infant characteristics**					
	Age at registration (weeks)^a^, mean (SD)	7.3 (3.6)	7.4 (3.6)	5.6 (3.4)	.02^d^
	Birth weight (kg)^a^, mean (SD)	3.46 (0.591)	3.47 (0.593)	3.47 (0.592)	.20
	Gender (male)^b^, n (%)	31 (55.3)	53 (46.9)	23 (41.0)	.34
**Baseline feeding status^c^, n (%)**					
	Breastfeeding	35 (63)	64 (56.6)	28 (50)	.13
	Formula feeding	14 (25)	25 (22.1)	23 (41)	
	Mixed feeding	7 (12.5)	24 (21.2)	5 (8.9)	

^a^Pearson correlation; mean, standard deviation (SD) reported.

^b^*t* test; % within group (count) reported.

^c^Based on ANOVA; % within group (count) reported.

^d^Statistically significant engagement level and independent variable <.05.

Of the 14 variables assessed in this study, 8 met the including criterion of *P* ≤.25 in the univariate analysis and were included in the multivariate linear model (full model) [26] presented in Table 2. Similar results were found for 4 variables which were significantly associated with EI scores as presented in the reduced model (see Table 2 for details). Higher EI scores were found among those mothers who were primiparous, using both the app and accessing the email, recruited to the program by their health practitioner and those who registered when their infant was younger.

**Figure 3:**
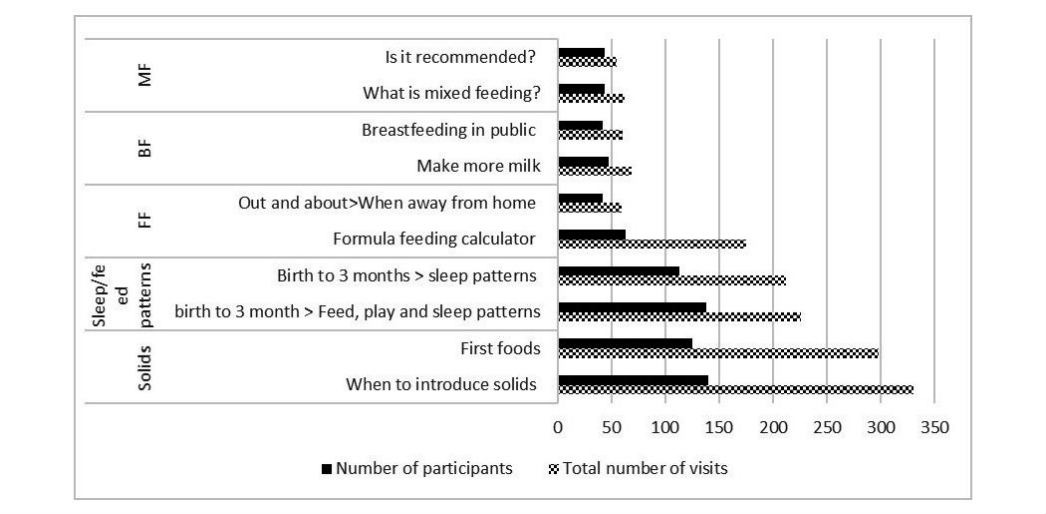
Number of participants and total number of times participants visited each section of the Growing healthy app. BF=breastfeeding, FF=formula feeding, MF=mixed feeding.

To better understand the drivers of engagement descriptive analysis of the subindices that made up the overall EI score was performed (Table 3). The click depth index (C_i_) median score was 30.8% (IQR: 21.0%-37.2%). Of the 303 pages that were available to view, the mean number of pages viewed was 30 (range: 1-156) and a median of 24. Although, throughout the program, participants viewed a mean of 44.2 pages (range: 1-316) and a median of 29. Figure 3 illustrates the most commonly viewed pages on the app including the number of times each page was visited and the number of participants that visited each page. The solids section was viewed the most and mixed feeding was viewed the least.

**Table 2:** Linear regression to explore the predictors of infant and participant characteristics with the engagement index scores.

Variable		Univariate model (B)	*P* value	Full model (B)	*P* value	Reduced model (B)	*P* value
R^2^				0.154		0.164	
**Parity**			.004		.006		.005
	Multiparous	1.00		1.00		1.00	
	Primiparous	4.532		4.147		4.209	
**Recruitment method**			.06		.07		.02
	Family or friends	1.00		1.00		1.00	
	Practitioner	5.346		6.423		4.221	
	Web-based	2.795		4.267		0.989	
**System type**			<.001		<.001		<.001
	App only	1.00		1.00		1.00	
	Both (app and email)	7.977		−6.426		−6.937	
	Infant age at T1 (weeks)	−0.477	.02	-0.522	.02	−0.459	.005
**Income**			.70				
	No response	1.00					
	Below Average	−0.033					
	Average	2.921					
	Above Average	0.061					
	Higher income	1.181					
**Marital status**			.59				
	Relationship	1.00					
	Single	2.208					
**Employment status**			.14		.08		
	Working or studying	1.00		1.00			
	Not in labor force	−3.189		−2.927			
**Country of birth**			.31				
	Other	1.00					
	Australia	−2.389					
	New Zealand	−0.074					
	United Kingdom	6.9.41					
**Device type**			.73				
	iOS	1.00					
	Android	0.580					
Birth weight (grams)		0.002	.20	0.001	.42		
**Gender**			.34				
	Male	1.00		1.00			
	Female	−1.462		−0.440	.77		
**Baseline feeding status**			.13		.17		
	Mixed feeding	1.00		1.00			
	Breastfeeding	−0.401		0.524			
	Formula feeding	3.124		3.941			

The loyalty index (L_i_) average score was 50.8% (IQR: 26.7%-75.7%). The average number of sessions participants visited the app was 11.6 times (range 1-64) and a median of 9. The recency index (R_i_) median score was 34.4% (IQR: 10.7%-37.3%). On average participants took 14 days to activate the app (range 0-184 days). The interaction index (I_i_) median score was 8.9% (IQR: 1.9%-18.1%). On average, 91.8 (range: 16-216) push notifications were sent and an average of 11.1 (range: 0-70) were opened with a median of 6. Participants who used both the app (including access to push notifications) and opened weekly emails scored lower on the I_i_ compared with participants who only used the app and only accessed push notifications.

The feedback index (F_i_) was calculated for 154 participants as 71 participants either did not complete the 9-month survey, or reported using the website (n=15) and were not asked for feedback about the app. The median score for F_i_ was 2.7 (IQR: 0-16.2). As presented in Table 4 the app features participants were most satisfied with included the language used, usefulness in sharing the app with another carer, and the quantity of Internet data required to use the app. Participants were least satisfied with the push notifications, including the number of push notifications sent (too few or too many), and many participants experienced technical problems using them. There was a low satisfaction with respect to the videos available on the app which they felt did not cover sufficient information to answer their queries about infant feeding.

**Figure 4:**
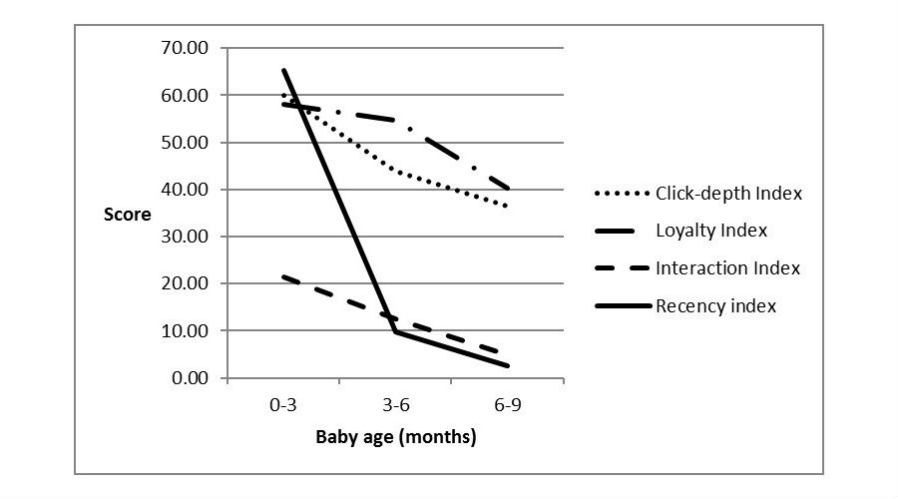
The frequency of scores for click-depth index (Ci), loyalty index (Li), interaction index (Ii), and recency index (Ri) at each time point (initial, interim, and final).

Over the duration of the program, there was a decrease in the mean index score for each subindex. The C_i_ and L_i_ scores shared similar scores during the initial (0-3 months) and final (6-9 months) period, whereas for the interim period (3-6 months) the mean score was lower for C_i_ (43.7%) compared with L_i_ (54.6%). The recency index dropped dramatically after the initial period by 55.4% and continued to track down, whereas the interaction index attained the lowest mean compared with the other subindices at the initial period (21.4%) and trended down over time (See Figure 4).

**Table 3:** Descriptive statistics of each subindex (N %).

Subindex	Mean	Median	Interquartile range	Range
Click depth index	46.7	45.5	33.3-63.3	0-100
Loyalty index	50.8	50.8	26.7-75.7	0-93.4
Recency index	26.0	34.4	10.7-37.3	0.6-53.7
Interaction index	12.7	8.9	1.9-18.1	0-64.3
Feedback index	13.3	2.7	0-16.2	0-94.6

**Table 4:** Participants’ reported satisfaction with aspects of the Growing healthy program (feedback index, F_i_; n=154).

Satisfaction questionnaire	Scores (N)^a^
I found the Growing healthy app easy to use	46
I liked the layout or “look” of the app	34
I found it hard to navigate through the app^b^	23
The Growing healthy app didn’t take long to load information	45
The Growing healthy app failed to work at times^b^	28
The different sections of the app worked well together	20
The language used in the app was easy to understand	57
The app did everything I expected it to do	31
I couldn’t find all of the answers I needed in the app^b^	11
I had to use the search feature to find what I was looking for	14
Using the app was an enjoyable experience	22
I found the app complicated^b^	43
I can trust the information on the Growing healthy app	39
I felt confident using this app	40
I found the information for mums useful	31
I found the information on feed and sleep patterns useful	29
I found the information about breastfeeding useful	20
I found the information about formula feeding useful	17
I found the information on mixed feeding useful	15
I found the information on solid feeding useful	27
I found the videos on the app useful	12
I found the recipe section of the app useful	22
I shared the information from the app with other friends and family	16
I was concerned about the Internet data usage on my phone when using the app^b^	47
I found the information provided easy to understand	36
Overall, I liked the Growing healthy program	36
I would recommend the Growing healthy program to a friend	45
I found it helpful to share the app with my partner or another carer	48
The Growing healthy program covered all of the things about infant feeding that I wanted it to	25
I received push notifications on my phone, from the Growing healthy program^c^	122
The push notification messages often disappeared before I had a chance to tap on them^b^	12
I didn’t know how to retrieve push notification messages once they disappeared from screen^b^	12
I would prefer to receive text messages rather than push notifications from the app	19
I was happy with the number of notifications or messages received each week	6
I was happy with the time that the notification was sent to me during the day	18
I found the notifications or messages helpful	16
I found the notifications or messages suited my baby’s age and stage of development	23

^a^Total scores only include the extreme positive responses based on scoring criteria **.**

^b^Likert scale scoring reversed for these questions: strongly disagree (1), disagree (0), no strong feelings either way (0), agree (0), strongly agree (1), and didn’t use (0).

^c^Response option and scoring: Yes, I received weekly push notifications (1), no, I received text messages instead of push notifications (1), and no, I disabled my push notifications so I didn't receive any weekly messages (0).

## Discussion

### Principal Findings

This is one of the first studies to develop and implement an mHealth program supporting parents with healthy infant feeding practices through a mobile phone app. To our knowledge, this is the first study to utilize an EI to quantify and categorize participants’ engagement level using the app. We found that engagement level was positively correlated with primiparous status, use of both the app and email, exposure to the program for a longer period, and recruitment through health practitioners. Negative correlation was found with age of child at start of program and engagement level.

The identification of the correlates of participant engagement is not only beneficial to inform future enhancements of the Growing healthy program, but more broadly to evaluate mHealth programs. The EI has its origins in measurement of consumer engagement with Web-based products. Adjusting the index to measure engagement with a mHealth program was possible as the metrics measured are the same; only the measurement of the content and behavior will be different [16].

A criterion to categorize participants as poor, moderate, or highly engaged with the Growing healthy program based on their overall EI score was developed. Previously, program engagement has arbitrarily been labeled as high [27] or low [22] based on the frequency participants accessed websites or apps. Few studies have considered participant engagement on the basis of their interaction with multiple intervention elements. In addition, there is not a standardized approach to measuring engagement. For example, a point system to gauge individual user activity with the program features was used in a study targeting reduction of high-risk sexual behaviors. The measures included were frequency of access, profile modification, message views, article views, completion of quizzes, number of pages viewed, and updates of personal goals [23]. Whereas modeling participants’ engagement with frequency of access, average daily steps, and the number of days since participants last accessed the program was performed for a physical activity focused mHealth intervention [20].

Participant app use over the 9-month period in this study varied such that engagement was high after initially joining the program but decreased from the 3- to 6-month period. Previous mHealth programs targeting long and short term behavior change have identified similar patterns of use [27-29]. Attrition with mHealth programs is negatively affected by factors such as lack of commitment or motivation to change health behaviors [28], confidence in knowledge about managing the targeted behavior [27], and programs that are perceived as overly burdensome by participants [23]. The Growing healthy program was a “just in time” resource [15] developed to provide infant feeding information up to 9 months of the infants age. Feeding milestones targeted included breastfeeding, best practice formula feeding, timing of the introduction of solids and optimizing dietary exposure to fruit and vegetables, and minimizing exposure to noncore foods. Once that knowledge is obtained, it is likely that participant app use will drop off [30]. When targeting long term behavior change, mHealth developers need to consider ongoing novel strategies that will keep participants engaged. Qualitative findings suggest users prefer to engage with apps periodically [28]. Such findings highlight that we must seek to understand app users’ behavior to inform the most appropriate time to engage them to join the program, the factors that lead to disengagement, and to consider strategies that will maintain their engagement.

Study participants who accessed both the website and the app attained a significantly higher EI score compared with participants who had just used the app. This supports the notion that delivering the intervention using various modes enhances engagement and to the intervention exposure [11,23]. Primiparous participants had significantly higher EI scores than multiparous women. This is congruent with qualitative analysis conducted as part of the development phase for the Growing healthy program (unpublished) where most of the primiparous participants expressed an interest in the program, while multiparous participants suggested the resource would have been more useful as a first time parent. Despite multiparous participants being less engaged, more than one third of those classified as highly engaged were indeed multiparous.

While initial engagement is the initial hurdle for any intervention, sustaining engagement remains the most difficult part of intervention implementation, it is more difficult to achieve [11,23,31]. It has been found that novelty and relevance are main contributors to sustained app user engagement [11,32,33]. The downward trend in engagement subindices scores from 3 to 6 months reported in this study may be a reflection of a lack of perceived novelty in the Growing healthy app throughout the intervention period. This may also explain the lower engagement of multiparous participants (who have developed their thinking around infant feeding already).

The infant’s age at baseline (ie, when the app was downloaded) was also strongly associated with higher EI scores. Participants who joined the program when their infant was younger had a higher EI score compared with those who joined when their infant’s age was closer to 3 months. Similar to traditional interventions that targeted childhood obesity prevention [34], early recruitment was necessary to increase participant engagement. Early recruitment is likely to increase intervention exposure, which is associated with an increased likelihood of influencing the uptake of the desired behaviors [27]. This is important to target as infant development clearly occurs rapidly within the first year of life. The app was likely to be most useful and provided novel information to mothers if they were recruited from early postpartum or during pregnancy.

Participants who were recruited from their health practitioner were more likely to have higher EI scores compared with those who were recruited on the Web. This may be attributed to mothers’ perception that health practitioners are a trustworthy source of information [35]. The involvement of health practitioners such as maternal and child health nurses and practice nurses who do routine infant health checks during the first few years of life [36], are important as a key “referral pathway” to evidence based apps and in turn, to the most effective utilization of apps.

### Comparison With Prior Work

Several studies describing mHealth interventions encouraging healthy infant feeding behaviors have recently been published. Delivery modes used in these studies included app [37,38], websites [39,40], and social media [41]. Due to the different delivery modes, the findings of this study cannot be compared with other programs. However, as mHealth interventions are novel modes of delivering health behavior change interventions across health disciplines, similar patterns of engagement have been reported by several researchers albeit using different measures [23,42,43].

### Limitations and Strengths

This study has several limitations. First, a number of technological issues were experienced by participants in receiving and opening push notifications. Adaptation were therefore made midway during the program and all participants were sent weekly emails. Second, app quality is an important influencer on participant engagement [44-46]. The participants’ responses to the satisfaction survey (feedback index) demonstrated low satisfaction with respect to the push notifications, emphasizing the impact technological difficulties have on participant engagement. Third, the weekly emails contained links to the Growing healthy website rather than the app. Finally, participant behavior on the website, such as the number of pages viewed, was not accessible at an individual level. This explains the increase in loyalty index scores at around 3 to 6 months, as participant access to email links was included. Click depth index scores decreased at that time point because the number of pages viewed on the website could not be measured. Overall, the EI score calculated for these participants is most likely an underestimate of their engagement with the program.

Some features of the Growing healthy program were not measured using the EI because there were difficulties in obtaining individual participants’ information such as, participant use of the Growing healthy Facebook group and sharing the app with another carer or sharing information from the app with others (interconnectivity). Although participant interaction with these features was not measured, satisfaction and use of these features was included in the 9-month survey that made up the feedback index.

Some studies have shown that mothers from a disadvantaged background were less likely to use the Internet as a source of information for infant feeding [47]. A strength of our study was that approximately equal number of participants of both high and low educational background were recruited unlike other mHealth programs targeted at addressing infant feeding [48].

To our knowledge, the utilization of an index to measure participant engagement has not yet been implemented in mHealth interventions. The EI provided detailed analysis regarding the frequency participants accessed the app and push notifications, how many pages they accessed per session, and their satisfaction with the program which was measured over 3 time points across the 9 months of the program.

### Conclusions

The EI provided a comprehensive understanding of participant behavior with the app over the 9-month period of the Growing healthy program. The participants’ engagement with the Growing healthy app was determined by various factors including participant characteristics, novelty, intervention exposure time, and the quality of the app including technological aspects. Primiparous participants, those who accessed both the emails and the app, those who were exposed to the program for a longer period, and those who were recruited from their health practitioner all had higher EI scores. The use of the EI in this study demonstrates that rich and useful data can be collected and used to assess the strengths and weaknesses of mHealth interventions and in turn inform improvements in their design and delivery.

## Appendix

Multimedia Appendix 1 Participant satisfaction survey.

## Abbreviations

Ci: Click depth index
EI: engagement index
Fi: feedback index
Ii: interaction index
Li: loyalty index
Ri: recency index
IQR: interquartile range
