# Supplementary figures and images for: Assessing User Engagement of an mHealth Intervention: Development and Implementation of the Growing Healthy App Engagement Index
Source: JMIR Mhealth Uhealth. 2017 Jun 29;5(6):e89. doi: 10.2196/mhealth.7236 (PMC5509951; doi:10.2196/mhealth.7236)

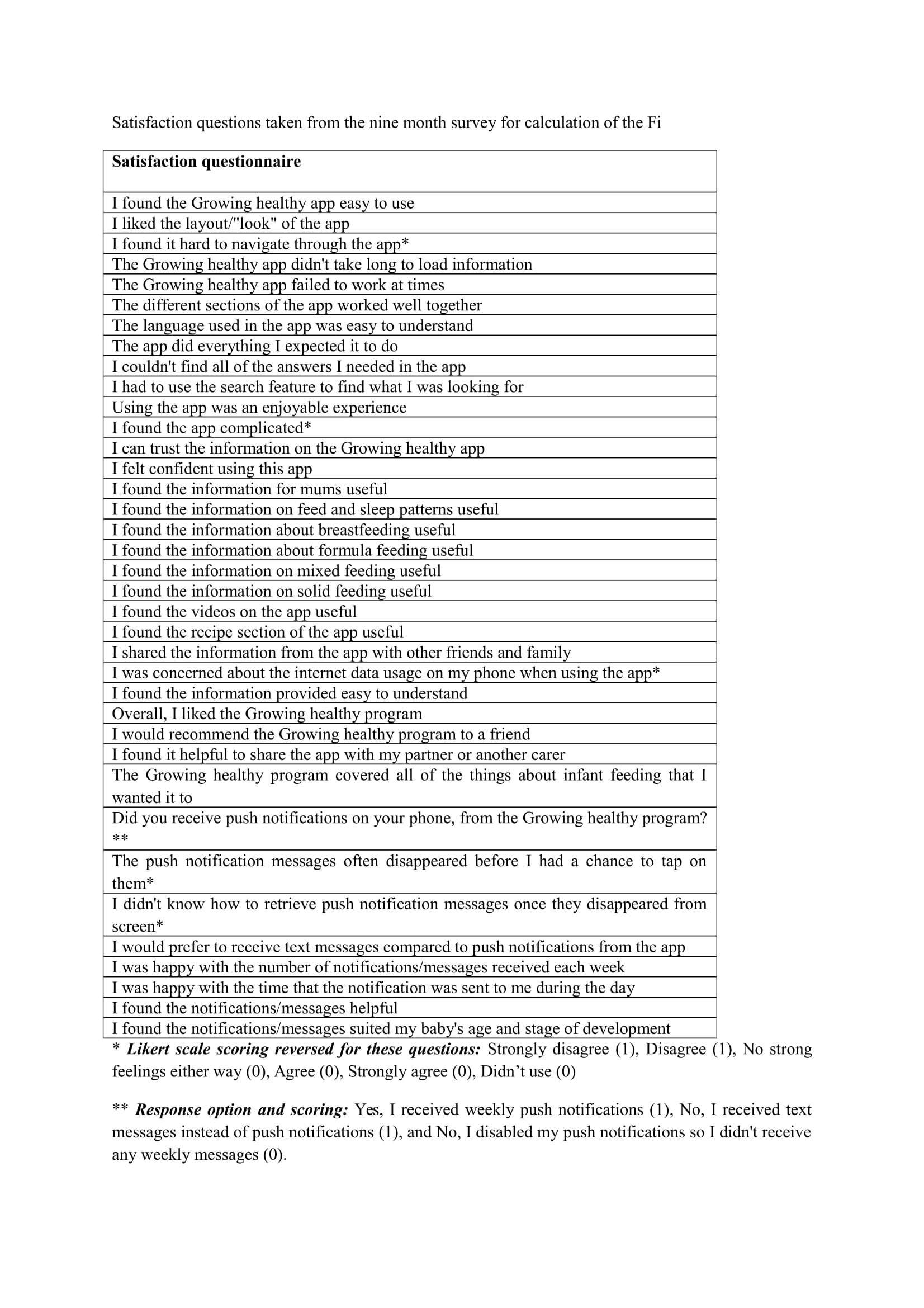

Supplement: Multimedia Appendix 1 [file mhealth_v5i6e89_app1.jpg]
